# Opposite Responses of Native and Nonnative Birds to Socioeconomics in a Latin American City

**DOI:** 10.3390/ani14020299

**Published:** 2024-01-18

**Authors:** Nélida R. Villaseñor, Catalina B. Muñoz-Pacheco, Martín A. H. Escobar

**Affiliations:** 1 Grupo de Ecología, Naturaleza y Sociedad, Departamento de Gestión Forestal y su Medio Ambiente, Facultad de Ciencias Forestales y de la Conservación de la Naturaleza, Universidad de Chile, Santiago 8820808, Chile; catalina.munoz.p@ug.uchile.cl (C.B.M.-P.); marescob@uchile.cl (M.A.H.E.); 2 Departamento de Ciencias Químicas y Biológicas, Universidad Bernardo O’Higgins, Av. Viel 1497, Santiago 8370993, Chile; 3 Escuela de Arquitectura del Paisaje, Universidad Central de Chile, Av. Toesca 1783, Santiago 8370292, Chile

**Keywords:** environmental justice, luxury effect, neotropical birds, urbanization, wildlife-friendly cities

## Abstract

**Simple Summary:**

To achieve sustainable cities that are sensitive to nature and environmentally just for urban dwellers is of global importance. For this, it is fundamental to understand the responses of native and nonnative species, identify the environmental variables that promote native species and limit nonnative species, and understand how they vary among socioeconomic groups. To help promote ecological justice and biodiversity conservation, we investigate the influence of socioeconomic level and woody cover on bird species richness and abundance in the Latin American city of Santiago de Chile. We also investigate whether bird response changes with bird origin (native vs. nonnative). We found that both socioeconomic level and woody vegetation cover influenced the bird community but had opposite effects on native and nonnative birds. Wealthier neighborhoods supported greater species richness and abundance of native birds. In contrast, poorer neighborhoods had greater bird abundance but were mainly composed of nonnative birds. Our study evidence showed that affluent neighborhoods provide more opportunities to encounter native birds and experience nature close to home than poorer neighborhoods. Given that woody cover positively influenced native birds and negatively influenced nonnative birds, increasing tree and shrub cover in neighborhoods where vulnerable people live will help support more native avifauna.

**Abstract:**

Due to the massive increase of the urban population, a global target is to achieve sustainable cities that are sensitive to nature and environmentally just for urban dwellers. To accomplish this, it is important to understand the responses of native and nonnative birds, identify the environmental variables that promote native species and limit nonnative species, and understand how they vary among socioeconomic groups. Although many cities in the Global South exhibit strong social and environmental segregation, few studies have investigated the relationship between socioeconomics and biodiversity. Therefore, to help promote ecological justice and biodiversity conservation in the developing world, we investigated the influence of socioeconomic level and woody cover on bird species richness and abundance in the city of Santiago de Chile. We also investigated whether bird response changes with species provenance—it is important to understand the response of native birds separately from nonnative birds because they imply opposite management strategies (e.g., conservation vs. species control/eradication). Thus, we surveyed 120 sites located in residential areas of high, medium, and low socioeconomic levels across the city and fit generalized linear (mixed) models that described bird species richness and abundance for total, native, and nonnative birds according to socioeconomic level and woody vegetation cover. We found that both socioeconomic level and woody vegetation cover influenced the bird community, but their effects changed with bird species origin, having opposite effects on native and nonnative birds. Residential areas where wealthier people live supported greater species richness and abundance of native birds than residential areas where people of lower socioeconomic status live. In contrast, residential areas where vulnerable people live had greater bird abundance that was mainly composed of nonnative birds. Therefore, affluent neighborhoods provide more opportunities to encounter native birds and experience nature close to home than poorer neighborhoods. Due to woody cover having positive effects on native birds and a negative influence on nonnative birds, increasing tree and shrub cover will contribute to supporting more native birds in residential areas deprived of woody vegetation, which are commonly low socioeconomic areas. Additional variables that can explain bird response among residential areas of different socioeconomic levels need to be investigated to better understand the factors influencing the distribution of birds in cities and promote a more biodiverse and environmentally just city.

## 1. Introduction

The number of people living on urban land has massively increased in the last century, emphasizing the need for achieving sustainable cities and towns. The number of humans living in urban settlements exhibited a 6-fold increase in the last 70 years, reaching 4.46 billion people in 2021, when an estimated 56% of the global population lived on urban land [1,2]. It is expected that by 2050 the urban population will reach 6.7 billion, and by that time, many of the humans will live in a city or town [2]. This increasing demand for urban land drives land use change and, thus, causes biodiversity decline and threatens several species with extinction [3]. In fact, urban areas expand faster than human population growth, impacting on important areas for biodiversity conservation [4], causing strong environmental change and the loss, degradation, and fragmentation of habitats [5,6]. Given current high rates of global biodiversity loss, it is important to plan, design, and manage urban landscapes to conserve biodiversity.

Biodiversity in cities is not only relevant to nature conservation but also for human societies. As most humans will live on urban land, biodiverse cities will offer daily opportunities for most people to experience nature close to home and work and to develop a meaningful relationship with nature [7,8]. Biodiversity in cities also provides multiple ecosystem services and contributes to human health and well-being [9,10,11,12]. For instance, Fuller et al. (2007) [10] reported that the psychological well-being of green space visitors increased with plant and bird diversity. In European cities, people prefer parks and streetscapes with higher species richness and feel that cities are more livable if they contain a high number of species [13]. Given that biodiversity in cities is relevant for human quality of life, a better understanding of the factors influencing the distribution of biodiversity in cities is required to ensure urban residents benefit from nature close to home and work.

Cities are heavily managed by humans, with different people actively selecting species by removing, introducing, or maintaining living organisms [14]. Therefore, biodiversity in cities might reflect social, economic, and cultural influences. For example, Hope et al. (2003) [15] found that plant diversity in high-income neighborhoods was twice that found in less wealthy areas in Phoenix, Arizona (USA). Thus, they proposed a positive relationship between human wealth and plant diversity in urban ecosystems, which they named the “luxury effect”. Since then, increasing evidence suggests that socioeconomics may be a good predictor not only for plant diversity but also for urban vegetation cover [9,16].

The inequity in the distribution of urban vegetation can also affect the distribution of fauna in cities. This is because the amount of urban vegetation can pose the strongest positive effects on animal diversity in urban landscapes [17,18]. Urban vegetation enhances habitat quality for wildlife, and it also increases landscape connectivity; thus, greater amounts of vegetation in urban areas contributes to supporting greater animal richness and abundance [19,20,21].

Although an increasing number of studies are investigating the luxury effect, most research has focused on plants, with few empirical studies on fauna [22] and the factors underpinning their response [23]. Past research has shown a positive relationship among human wealth (income) and bird species richness in urban parks, residential areas [24], and municipal districts [25]. However, this relationship might be different in developing countries [26] or change depending on bird species provenance (e.g., native species might respond differently than nonnative species) [27].

To date, most research on the relationship between human wealth and biodiversity has been performed in more developed countries of the Northern Hemisphere, with few empirical studies in the developing world [22,28,29]. Given that most future urban growth is predicted to occur in developing countries in the next decades [30], it is important to understand the relationship between human wealth and biodiversity to promote sustainable and environmentally just urban development in these countries. In addition, few studies have investigated whether the relationship between wealth and bird diversity changes with species provenance [29]. This is an important gap in the research that limits management strategies associated with biodiversity conservation in cities and towns.

To help promote ecological justice and biodiversity conservation in the developing world, we investigated the influence of the socioeconomic level and woody cover on bird species richness and abundance in Santiago de Chile. Santiago is a large Latin American city with a Mediterranean climate that exhibits strong environmental segregation. We also investigated whether bird response changes with species provenance, because nonnative birds commonly increase with the built environment, and, in contrast, native birds tend to reach high species richness and abundance in urban areas that present greater vegetation cover [21,31]. Understanding the response of native birds separately from nonnative birds is important due to their opposite management implications (conservation vs. species control/eradication). Therefore, the empirical evidence provided by this study will aid in understanding the relationship between socioeconomics and bird diversity, contributing to making informed decisions associated with native and nonnative birds, as well as promoting a more equitable city where all residents can experience nature close to home and work.

## 2. Materials and Methods

### 2.1. Study Area

The study was performed in Santiago de Chile (33°27′ S–70°40′ W), the country’s capital city. Santiago is the largest (650 km^2^) and most populated (>6 million inhabitants) city in Chile and one of the most populated cities in Latin America [32]. Situated in Central Chile, this region encompasses ecosystems rich in endemic species. However, the landscape has undergone significant alteration and conversion caused by human activities, and, given the loss of the majority of natural ecosystems, Central Chile has been identified as a global biodiversity hotspot [33]. The climate exhibits a temperate Mediterranean pattern, featuring warm and dry summers (December to March) with temperatures reaching up to 35 °C and cool winters (June to September) marked by lower temperatures reaching −3 °C. While the annual precipitation hovers around 260 mm, a large drought has extended for more than ten years [34]. This drought has led to substantial rainfall deficits ranging from 20% to 40% [35].

Santiago is a city that exhibits strong social segregation, as well as an uneven distribution of green space [36]. People of high income aggregate in the northeastern part of the city, which is also the urban area in the city with the greater vegetation cover. In fact, neighborhoods where people of higher income levels live commonly have larger green space area, greater woody cover, and greater diversity of plants than neighborhoods where people of lower income levels live [37,38,39].

### 2.2. Selection of Study Sites

**Figure 1:**
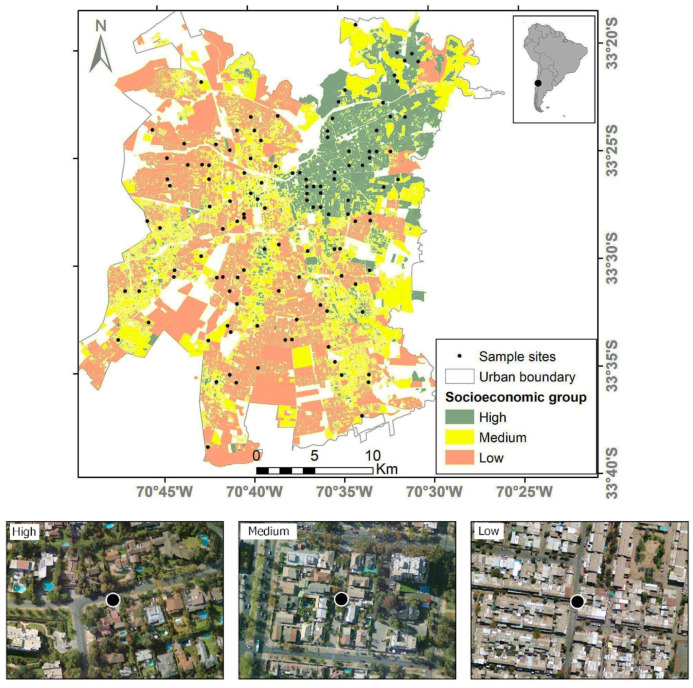
Sample sites and socioeconomic groups in the city of Santiago de Chile. For each socioeconomic group, an example of a sample site is provided using satellite imagery from Google Earth. Modified from [23].

We used 120 sites located in residential areas of different socioeconomic levels selected by Villaseñor and Escobar (2022) [23]. To select sites, these authors used a digital layer of the socioeconomic distribution [40]. These socioeconomic levels were summarized into three main groups: high, medium, and low. The high socioeconomic group was the wealthier, university-educated group, with a mean annual household income greater than US 28,800. The medium socioeconomic group comprised households with technical or secondary education and a mean annual household income higher than US 13,200. The low socioeconomic group comprised households with less education and a mean annual household income lower than US 8400 [41]. Using the “raster” package [42] in R 4.1.2 [43], this layer was rasterized at a 200 m × 200 m cell size to generate a grid of potential sample sites [23]. Because previous research in Santiago describes a decrease in species richness towards the interior of the city [21], the city was stratified in two areas: edge (less than 5 km from the urban limit) and interior (more than 5 km from the urban limit towards the interior of the city). A stratified random selection approach was used to select 20 sites (raster cells) in residential areas for each combination of socioeconomic level (three levels: high, medium, and low) and distance to the urban boundary (two levels: edge and interior), leading to 120 sites (Figure 1; [23]).

### 2.3. Bird Surveys

Birds were surveyed twice at 120 sites during the austral breeding season (from 12 October to 4 November 2021) to reduce bias associated with particular conditions of a given day within the season. Two surveys per season provide a reliable indication of bird trends and have successfully described bird community response in our city [21,23,31]. At each site, the point count method was used to record all birds seen or heard during 5 min within a visually estimated 50 m radius plot [44] but excluding birds flying over that did not use the area [21]. To reduce observer bias, two different observers surveyed all sites, who sampled the same site on different days. The time interval between bird surveys performed in the same site ranged from 3 to 15 days (mean = 11 days, SD = 2.7 days). All counts were performed during mornings (from 6:40 am to 11:45) and on days with no rain, fog, or strong wind [23].

### 2.4. Woody Vegetation Assessment

For each sampling site, we calculated the percent cover of woody vegetation in 50 m radius plots—the area comprised by bird counts. We interpreted woody cover in 50 m radius plots because they are effective at characterizing habitat conditions and commonly show significant relationships with birds in our study area [18,21,31]. Thus, in ArcGIS we created 50 m radius plots that had their center in the same location of the bird point count [23]. Then, high-resolution satellite imagery (WorldView, DigitalGlobe) was visually interpreted and digitalized within each plot to later calculate the percent area covered by woody vegetation.

### 2.5. Statistical Analyses

For each site, we calculated cumulative species richness and average abundance of total, native, and nonnative birds from the two counts. Native and nonnative species were classified following [45], except for shiny cowbird (*Molothrus bonariensis*) because of its uncertain origin [46]. To investigate whether cumulative species richness and average abundance of these three bird groups (total, natives, and nonnatives) varied with socioeconomic level, we fitted a generalized linear model (GLM) with a Poisson distribution (link = log) in R. We also included in the models the additive effect of the percent woody cover in 50 m radius plots to investigate the influence of woody cover on birds.

Thus, all predictive models included socioeconomic level (factor with three levels: high, medium, and low) and percent woody cover as fixed effects. Fitted models were assessed for overdispersion and underdispersion. When a model was overdispersed, we fitted generalized linear mixed models to include an observation-level random effect using the “lme4” package [47]. When a model was underdispersed, we used the COMPoisson distribution with the “spaMM” package [48]. After examining model residuals, we interpreted variable estimates and calculated and plotted confidence intervals at 95%.

We used R 4.2.0 for all statistical analyses.

## 3. Results

During the austral breeding season, we obtained a total of 2953 bird records in 120 sites that were surveyed twice. They comprised 31 bird species, including 26 native species (1262 records), four nonnative species (1649 records), and one species of uncertain origin (*M. bonariensis*, with 42 records). Three nonnative species accounted for 56% of the total bird records: feral pigeon (*Columba livia*, 28.8% of records), house sparrow (*Passer domesticus*, 21.3% of records), and monk parakeet (*Myiopsitta monachus*, 5.6% of records). The most abundant native species comprised eared dove (*Zenaida auriculata*, 12.5% of records), austral thrush (*Turdus falcklandii*, 8.6% of records), and rufous-collared sparrow (*Zonotrichia capensis*, 6% of records; Table A1 in Appendix A). Woody vegetation cover ranged from 0–38%, with an average value of 10% at the 50 m radius plots.

**Figure 2:**
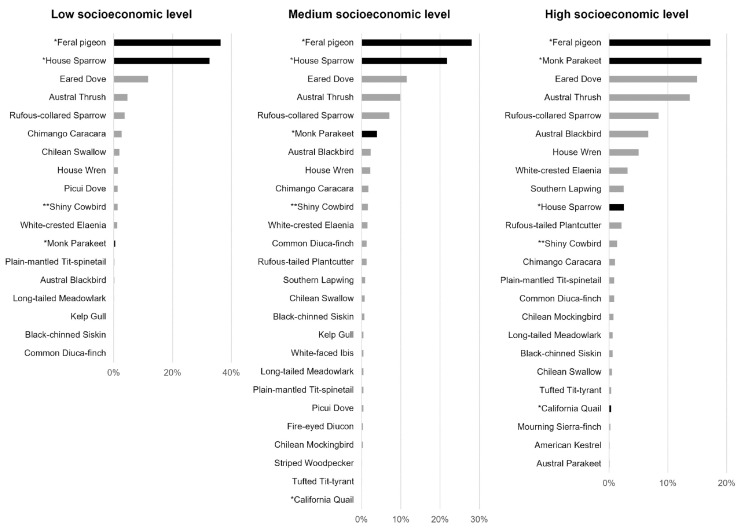
Relative abundance of bird species recorded in residential areas of low, medium, and high socioeconomic levels. Asterisks show nonnative species (*, black bar) and species of uncertain origin (**).

Nonnative species reached the highest number of records in residential areas across all socioeconomic levels, although they strongly dominated the bird community in low socioeconomic areas (Figure 2; Supplementary Material Figure S1). Among 18 species recorded in residential areas of low socioeconomic level, the most abundant were *C. livia* (36% of records) and *P. domesticus* (33% of records). Among 26 species recorded in residential areas of medium socioeconomic level, the most abundant were *C. livia* (28%) and *P. domesticus* (22%). Among 24 species recorded in residential areas of high socioeconomic level, the most abundant were *C. livia* (17% of bird records) and *M. monachus* (165%), followed closely by native species *Z. auriculata* (15%) and *T. falckandii* (14%; Figure 2).

**Figure 3:**
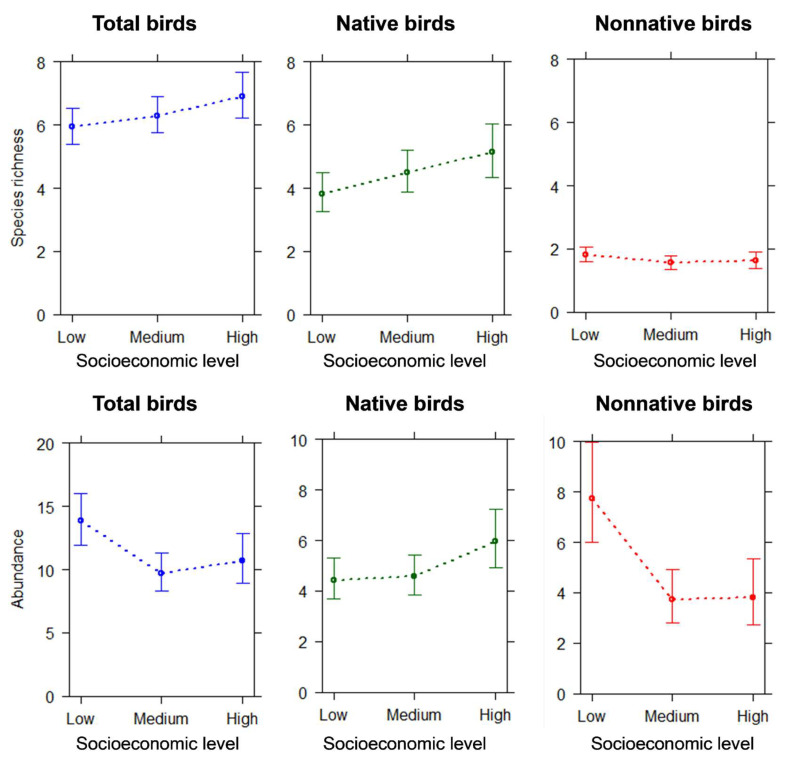
Results from generalized linear (mixed) models predicting species richness and average abundance of total, native, and nonnative birds according to neighborhood socioeconomic level. Error bars represent 95% confidence intervals.

**Figure 4:**
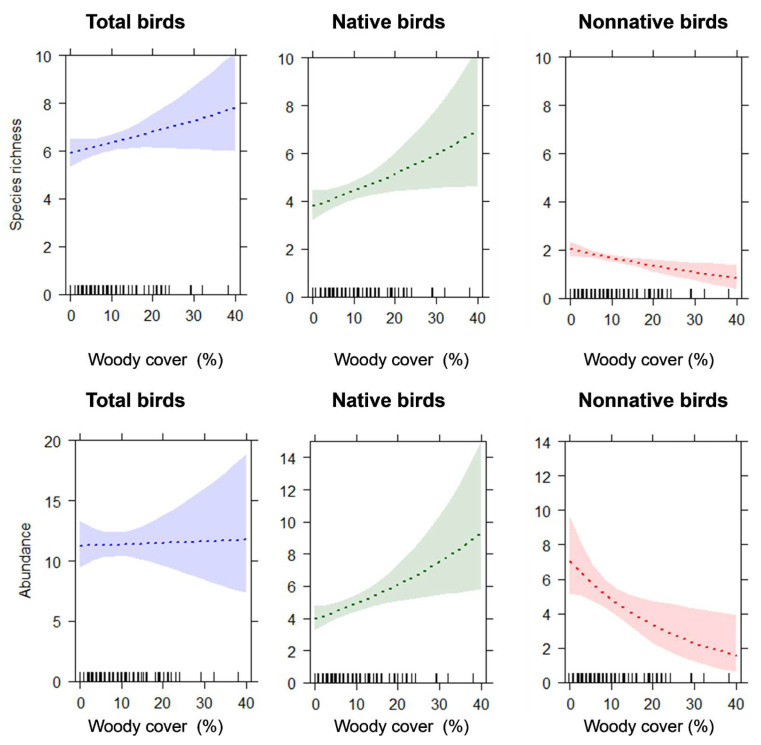
Results from generalized linear (mixed) models predicting species richness and average abundance of total, native, and nonnative birds according to percent woody cover in 50 m radius plots. Shadow areas represent 95% confidence intervals.

The statistical models showed that bird species richness and abundance significantly varied with neighborhood socioeconomic level (Table 1, Figure 3). The total bird species richness was significantly higher in sites located in residential areas of high socioeconomic level than those of low socioeconomic level (*p* = 0.049). While native species richness was higher in residential areas of high socioeconomic level than in low socioeconomic level (*p* = 0.02), nonnative species richness exhibited greater values in residential areas of low socioeconomic level, although differences were not significant with respect to residential areas of medium socioeconomic level (*p* = 0.08). Woody cover did not exhibit a statistically significant effect on total species richness; however, woody cover exhibited a positive effect on native species richness (*p* = 0.03) and a negative effect on nonnative species richness (*p* = 0.004; Table 1, Figure 4).

The total bird abundance was significantly higher in residential areas of low socioeconomic level compared to both medium and high socioeconomic levels (*p* < 0.001 and *p* = 0.04, respectively). While native birds were more abundant in residential areas of high socioeconomic level compared to low level (*p* = 0.03), nonnative species were more abundant in residential areas of low socioeconomic level compared to both medium and high levels (*p* < 0.001 and *p* = 0.002, respectively). Woody cover did not exhibit a significant effect on the total bird abundance (*p* = 0.9). However, it exhibited a positive effect on the abundance of native birds (*p* = 0.007) and a negative effect on the abundance of nonnative birds (*p* = 0.1; Table 1, Figure 4).

## 4. Discussion

Due to the massive increase of the urban population, a global target is to achieve sustainable cities and towns that are sensitive to nature and environmentally just for urban dwellers [49]. To achieve this, it is important to understand the different responses of native and nonnative birds, identify the environmental variables that promote native species and limit nonnative birds, and understand how they vary among socioeconomic groups. Our study reveals that socioeconomic level and woody vegetation influence the bird community, but their effects change with the origin of bird species, having opposite effects on native and nonnative birds. Residential areas where wealthier people live support greater species richness and abundance of native birds than residential areas where people of lower socioeconomic status live. In contrast, residential areas where vulnerable people live have greater bird abundance that is mainly composed of nonnative birds. Therefore, affluent neighborhoods provide more opportunities to encounter native birds and experience nature close to home than poorer neighborhoods. Due to woody cover having positive effects on native birds and a negative influence on nonnative birds, increasing tree and shrub cover will contribute to the support of more native birds in residential areas deprived of woody vegetation. Additional variables that can explain bird response among residential areas of different socioeconomic levels need to be investigated to better understand the factors influencing the distribution of birds in cities and promote a more biodiverse and environmentally just city.

### 4.1. Socioeconomics, Woody Cover, and Native Birds

Bird species richness increased with neighborhood socioeconomic level, supporting the “luxury effect”, which describes a positive relationship between human wealth and species richness in cities [15]. This positive effect of socioeconomics on bird species richness was underpinned by the response of native birds. There is a consistent pattern of positive correlations between neighborhood income and bird species richness, with such associations being frequently documented in North America, Europe, and New Zealand [24,25,27,50,51,52]. In addition, we found that native bird abundance increases with neighborhood socioeconomic level, which agrees with previous findings of greater native bird abundance in wealthier areas in a southern city [53].

Although different studies have found greater bird diversity at high income levels [22], the results are less clear in developing countries [29]. For example, in developing South Africa, neighborhood income had no effect on native bird species richness due to the presence of a large number of wetlands in poorer neighborhoods, which hosted a large number of species [54]. In contrast, in Latin America, several cities exhibit strong social and environmental segregation, with poorer people having less access to greenspace and biodiversity [39,55]. Given that developing regions commonly lack policies that promote environmental justice, the luxury effect is likely stronger in these countries than in more developed ones [22]. New studies on the relationship between urban wildlife and human wealth need to be conducted in developing countries to improve our understanding of the relationship between socioeconomics and animal diversity.

We found a strong positive effect of woody vegetation cover on species richness and the abundance of native birds, which is consistent with our previous findings [21,56]. Urban trees and shrubs provide several resources for native birds in our study area, including food supply, resting, and nesting sites [57,58]. Although urban trees and shrubs are an important attribute in urban environments, providing key habitat structures for birds and several ecosystem services for people, they are not equally distributed across the city. People of greater socioeconomic status commonly live in greener neighborhoods, with greater woody cover and tree diversity [39,59]. Thus, this inequity in the distribution of urban vegetation between socioeconomic groups leads to differences in the distribution of native birds [18,23].

In this investigation, we found a positive effect of socioeconomics on the richness and abundance of native birds in addition to the positive effect of woody vegetation cover on native birds. Previous research in our study area provides evidence that native bird occurrence and species richness increase with socioeconomic level, and this effect is mediated by greater woody vegetation cover in wealthier areas [18,23]. However, socioeconomics is likely linked to several variables that can be relevant for birds, including important habitat attributes, such as local garden management (e.g., small-scale vegetation attributes [15,60]) and food supply. For instance, urban bird feeders are more common in neighborhoods with a higher socioeconomic level [10]. The abundance of bird predators such as free-roaming cats and dogs decreases with increasing socioeconomic level [18]. Therefore, further research is needed to better understand the mechanisms by which socioeconomics influence bird diversity.

### 4.2. Socioeconomics, Woody Cover, and Nonnative Birds

The response of nonnative birds was opposite to that observed in native birds, since the abundance of nonnative birds decreased with increasing socioeconomic level. In fact, low socioeconomic neighborhoods exhibited the highest abundance of nonnative birds. This finding is consistent with previous research on nonnative birds in our country, where the feral pigeon (*C. livia*) and the house sparrow (*P. domesticus*) reach their greatest abundance in low socioeconomic neighborhoods [53,61]. Few studies have assessed the relationship between socioeconomic status and nonnative bird diversity [22]; however, in Chicago (USA), no relationship was identified between per capita income and nonnative bird abundance [27].

We did not find a significant effect of socioeconomics on nonnative species richness. Contrary to our results, a positive association has been proposed between human wealth and nonnative species that have established self-sustaining populations in urban environments [29]. For example, a study carried out in Chicago (USA) showed that neighborhoods with high per capita income had more nonnative species than neighborhoods with lower income and documented six nonnative bird species [27]. In our study, the lack of statistically significant results on nonnative species richness between socioeconomic groups may be the result of a low number of nonnative bird species in our city. In Santiago, only three species are widely distributed across the city: *C. livia f. domestica*, *P. domesticus*, and *M. monachus* [61].

Unlike native birds, we found that the species richness and abundance of nonnative birds decreased with increasing woody vegetation cover. This result is consistent with previous findings at the species level, where the abundance of nonnative species *P. domesticus* and *C. livia* decrease with increasing woody vegetation [31]. Built-up areas commonly offer appropriate habitats for these species, providing anthropogenic food and nesting sites, allowing these birds to reach high abundance [31,52,62]. In addition, woody vegetation promotes greater abundance and richness of native birds [21,56] that may interact with nonnative birds through competition [63,64], limiting the abundance of nonnative birds.

### 4.3. Origin of Species and Masking Effects

Most studies have found a consistent pattern of increasing bird diversity in areas of high socioeconomic level [22], although some studies have also reported negative and neutral effects [26,27]. Neutral outcomes could stem from compensatory relationships between native and nonnative species, wherein their influences counterbalance each other [27]. Here we call this phenomenon a “masking effect”.

In our study, the total bird richness increased with neighborhood socioeconomic level, a pattern that was underpinned by the richness of native species. A greater number of native species (26 species) than nonnative birds (4 species) was recorded in the city, evidencing the larger native species pool available, and this differed among socioeconomic groups. Given that only four nonnative species were recorded, differences in nonnative species richness between socioeconomic levels were less likely to be statistically significant and, thus, no masking effect on the total bird species richness was found.

In contrast, the total bird abundance was significantly higher in neighborhoods of low socioeconomic level, a pattern underpinned by nonnative birds. In particular, two species (*P. domesticus* and *C. livia*) are widely distributed in the city and are vastly more abundant in residential areas of low socioeconomic level compared to the medium and high levels [60]. This result completely masks the opposite pattern exhibited by native birds, which were more abundant in residential areas of high socioeconomic level compared to those of low socioeconomic level.

The masking of the opposite responses of native and nonnative birds was more evident in the case of woody cover, which did not show significant effects on either the total species richness nor the total bird abundance, despite presenting positive effects on native birds and negative effects on nonnative birds.

Our findings highlight the need to study animal responses according to their origin in urban ecological studies [27,65], since native and nonnative species are subject to different management and planning goals. While native species are subject to conservation actions, nonnative birds can be subject to control strategies to reduce or eradicate their populations. Therefore, management measures implemented from research results that do not differentiate species origin could even be counterproductive to the conservation of native species. Unfortunately, most studies on urban bird ecology in South America do not analyze bird communities according to their origin, a trend that is worrying due to the potential masking effects or bird community patterns reflecting mostly the response of nonnative birds.

### 4.4. Recommendations

Sustainable urban development is one of the United Nations Sustainability Goals (2018) [66]; therefore, it is important to achieve cities that both allow human prosperity, in that they are environmentally fair for urban residents, and promote the conservation of nature [49]. For this, there are several recommendations that can be derived from our work.

First, when examining community responses, we recommend investigating separately the response of native and nonnative birds. This is due to these birds being subject to different management actions (conservation vs. control), and they can exhibit different responses to both social and environmental variables. Native and nonnative birds exhibited opposite responses to both socioeconomics and woody vegetation cover. Therefore, and depending on the magnitude of the effect on each group, if researchers examine the results aggregated for the entire bird community, it can lead to neutral responses (no effect) or reflect the response of only one group.

Second, we found evidence of a luxury effect, due to bird species richness increasing with neighborhood socioeconomic level. Given that our city exhibits a luxury effect, people of lower socioeconomic status have fewer opportunities to interact with a variety of native birds than people of greater socioeconomic status. This can lead to more vulnerable people being more isolated from nature, which highlights the need to develop policies to address this injustice and ensure that all people, independent of their socioeconomic status, can access and receive benefits from biodiversity. Furthermore, it is important that governments address inequalities in access to nature in cities, because these differences are reported to increase over time [67].

Third, due to the positive effect of woody cover on both the richness and abundance of native birds, management actions that aim to increase tree and shrub cover in neighborhoods where people of lower socioeconomic status live could help to reduce environmental differences among socioeconomic groups and increase native birds [21,23,56]. Increasing woody cover in residential areas of low socioeconomic level will also aid in controlling the abundance of nonnative birds widely distributed in the city [31]. Thus, this management action will contribute to addressing inequalities among neighborhoods of different socioeconomic status, promoting equitable access to biodiversity and a more environmentally just city.

## 5. Conclusions

Our study reports that socioeconomic level and woody vegetation cover influenced the bird community but had opposite effects on native and nonnative birds. When analyzing the response of total birds (without considering different origin), nonnative birds produced a “masking effect” on native bird patterns. Therefore, it is important to take into account species provenance in urban ecological studies, since native and nonnative species are subject to different management and planning objectives.

Residential areas where wealthier people live supported greater species richness and abundance of native birds. In contrast, residential areas where vulnerable people live had higher bird abundance, which was primarily composed of nonnative birds. Since wealthy neighborhoods provide more opportunities to find native birds and experience nature close to home than poorer neighborhoods, this ecological injustice needs to be addressed by improving habitat quality in more vulnerable neighborhoods. A management strategy that increases woody cover in these neighborhoods will likely achieve positive outcomes, as tree and shrub cover will promote greater species richness and abundance of native birds.

Finally, additional research should be conducted to identify environmental variables that promote native species and limit nonnative species in urban areas, particularly in developing countries in the Global South, to help promote more biodiverse and environmentally just cities.

## Figures and Tables

**Table 1:** Estimates from generalized linear (mixed) models predicting species richness and average abundance of total, native, and nonnative species (from two counts). For total and nonnative species richness, results are from GLMs with COMPoisson distribution; for native species richness, results are from GLM with Poisson distribution; for total, native, and nonnative bird abundance, results are from GLMMs with Poisson distribution (see Section 2. Methods).

Response Variable	Variables	Estimate	Std. Error	*p*
Total species richness	Intercept	3.52	0.10	<0.001
	Socioeconomic_Medium_	0.11	0.13	0.37
	Socioeconomic_High_	0.29	0.15	0.05
	Woody cover	0.01	0.01	0.12
Native species richness	Intercept	1.20	0.09	<0.001
	Socioeconomic_Medium_	0.16	0.11	0.14
	Socioeconomic_High_	0.29	0.13	0.02
	Woody cover	0.02	0.01	0.02
Nonnative species richness	Intercept	3.82	0.23	<0.001
	Socioeconomic_Medium_	−0.50	0.28	0.08
	Socioeconomic_High_	−0.36	0.36	0.32
	Woody cover	−0.07	0.02	0.004
Total bird abundance	Intercept	2.62	0.09	<0.001
	Socioeconomic_Medium_	−0.35	0.11	<0.001
	Socioeconomic_High_	−0.26	0.13	0.05
	Woody cover	0.001	0.01	0.88
Native bird abundance	Intercept	1.28	0.10	<0.001
	Socioeconomic_Medium_	0.03	0.12	0.80
	Socioeconomic_High_	0.30	0.14	0.03
	Woody cover	0.02	0.01	0.01
Nonnative bird abundance	Intercept	2.41	0.15	<0.001
	Socioeconomic_Medium_	−0.73	0.18	<0.001
	Socioeconomic_High_	−0.71	0.23	0.002
	Woody cover	−0.04	0.02	0.01

## Data Availability

The data presented in this study are available on request from the corresponding author. The data are not publicly available due to it is part of an ongoing research.

## Supplementary Materials

The following supporting information can be downloaded at https://www.mdpi.com/article/10.3390/ani14020299/s1. Figure S1. Boxplots of species richness and abundance for total, native, and nonnative birds by socioeconomic level. Points are the observed data values.

## Appendix A

**Table A1:** List of species recorded during the study. Their origin, abundance, and total records are shown. Species are ordered according to their abundance. (*) Sighting of the species outside the sampling count (not used for analyses).

Common Name	Scientific Name	Origin	Abundance(Ind/Count)	Total Records
Feral Pigeon	*Columba livia*	Nonnative	3.56	850
House Sparrow	*Passer domesticus*	Nonnative	2.64	630
Eared Dove	*Zenaida auriculata*	Native	1.54	369
Austral Thrush	*Turdus falcklandii*	Native	1.06	254
Rufous-collared Sparrow	*Zonotrichia capensis*	Native	0.74	176
Monk Parakeet	*Myiopsitta monachus*	Nonnative	0.69	165
House Wren	*Troglodytes aedon*	Native	0.32	77
Austral Blackbird	*Curaeus curaeus*	Native	0.32	76
Chimango Caracara	*Daptrius chimango*	Native	0.24	58
White-crested Elaenia	*Elaenia albiceps*	Native	0.22	53
Shiny Cowbird	*Molothrus bonariensis*	Uncertain	0.18	42
Chilean Swallow	*Tachycineta leucopyga*	Native	0.15	36
Southern Lapwing	*Vanellus chilensis*	Native	0.12	28
Rufous-tailed Plantcutter	*Phytotoma rara*	Native	0.12	28
Picui Dove	*Columbina picui*	Native	0.09	21
Common Diuca-finch	*Diuca diuca*	Native	0.08	19
Plain-mantled Tit-spinetail	*Leptasthenura aegithaloides*	Native	0.06	14
Black-chinned Siskin	*Spinus barbatus*	Native	0.05	12
Long-tailed Meadowlark	*Leistes loyca*	Native	0.05	11
Chilean Mockingbird	*Mimus thenca*	Native	0.04	9
California Quail	*Callipepla californica*	Nonnative	0.02	4
Tufted Tit-tyrant	*Anairetes parulus*	Native	0.02	4
White-faced Ibis	*Plegadis chihi*	Native	0.02	4
Kelp Gull	*Larus dominicanus*	Native	0.02	5
Fire-eyed Diucon	*Pyrope pyrope*	Native	0.01	3
Mourning Sierra-finch	*Rhopospina fruticeti*	Native	0.01	2
Austral Parakeet	*Enicognathus ferrugineus*	Native	0.004	1
Striped Woodpecker	*Veniliornis lignarius*	Native	0.004	1
American Kestrel	*Falco sparverius*	Native	0.004	1
Harris’s Hawk	*Parabuteo unicinctus*	Native	*	
Cinereous Harrier	*Circus cinereus*	Native	*	
