# Supplementary material for: Opposite Responses of Native and Nonnative Birds to Socioeconomics in a Latin American City
Source: Animals (Basel). 2024 Jan 18;14(2):299. doi: 10.3390/ani14020299 (PMC10812454; doi:10.3390/ani14020299)

Supplementary Material for the article:

Opposite Responses of Native and Nonnative Birds to Socioeconomics in a Latin American City

Nélida R. Villaseñor, Catalina B. Muñoz-Pacheco & Martín A. H. Escobar

Figure S1. Boxplots of species richness and abundance of total, native and nonnative birds by socioeconomic level. Points are the observed data values.

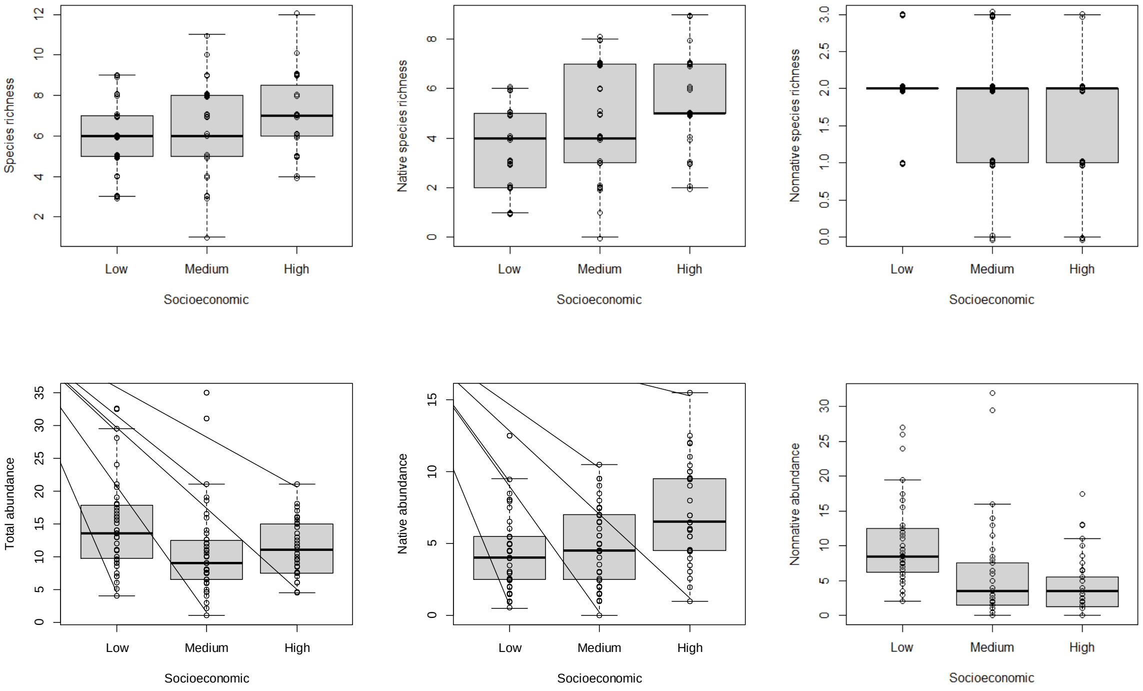

Supplement: Supplementary file 1 [file animals-14-00299-s001.zip › animals-2801756-supplementary.pdf]
